# The Multidimensional Factors That Influence the Family Management of Autism Spectrum Disorder: A Mixed Methods Study

**DOI:** 10.1177/10748407251333201

**Published:** 2025-05-12

**Authors:** Shayleigh Dickson Page, Kylie Trone, Margaret C. Souders, Jennifer A. Pinto-Martin, Janet A. Deatrick

**Affiliations:** 1 University of Pennsylvania, Philadelphia, USA; 2 Children’s Hospital of Philadelphia, PA, USA

**Keywords:** family management, autism spectrum disorder, mixed methods

## Abstract

Children with autism spectrum disorder (ASD) have complex health needs and co-occurring medical and psychiatric diagnoses. Using the Family Management Style Framework, this convergent parallel mixed methods (QUAN + qual) study: (a) examined the intersection of Ability and Effort to define family management patterns and (b) evaluated the influence of child (ASD-related behaviors, feeding difficulties, sleep disturbances, gastrointestinal symptoms, aggression, self-injury), caregiver (anxiety, depression), and family (social support, unmet social needs) factors on family management pattern. Fifty-six primary caregivers of children with ASD completed the quantitative strand of the study. A nested sample of 30 caregivers participated in semi-structured interviews. The four patterns of family management were similar to those previously identified. Data from quantitative measures and interviews converged to identify that specific child characteristics (ASD behaviors, sleep disturbances, aggression, self-injury) and the caregiver’s perceived social support influence family management. Descriptions of family management patterns and their correlates are important to guiding family nursing for this population.

## Introduction

Autism spectrum disorder (ASD) is characterized by neurodevelopmental differences that are inclusive of diverse abilities and challenges. Diagnostic criterion for ASD includes differences in social communication and restricted, repetitive patterns of behavior that are present across the lifespan (American Psychiatric Association, 2013). These neurodevelopmental differences can make participation in school and home activities more difficult for children with ASD compared to children with typical neurodevelopment. ASD can also be more broadly conceptualized under the umbrella of neurodiversity (Singer, 2017), whereby differences in learning and behavior are not inherent deficits. Research to date on ASD and families has documented numerous impacts of an ASD diagnosis on the family, including that caregivers experience greater stress (S. A. Hayes & Watson, 2013), family members have poorer mental health (Cohrs & Leslie, 2017; Shivers et al., 2019), and the functioning of the family unit is more disrupted (Sikora et al., 2013). Siblings of children with ASD are at increased risk for internalizing behavior problems and experiencing symptoms of depression and anxiety (Shivers et al., 2019). The unpredictability and intensity of behaviors associated with ASD can lead to isolation from extended family and social networks as well as the inability to engage in leisure activities as a family (Kirkpatrick et al., 2019; Myers et al., 2009). However, not all impacts on the family are negative and qualitative data has provided insight into the positive impacts of ASD on family members. For example, siblings have reported that having a brother or sister with ASD made them more empathetic and compassionate toward others, though it took time to understand ASD and adjust to differences in family life (Leedham et al., 2020). Qualitative research has also identified that caregivers often describe co-existing and often conflicting emotions (e.g., joy and heartache, hope and fear) when speaking about their child and family (Larson, 1998; Myers et al., 2009). Critical to moving the science forward is a deeper understanding of modifiable factors that can strengthen the family’s approach to caring for the child’s complex developmental, physical, emotional, and behavioral health needs.

The Family Management Style Framework (FMSF) outlines the responses of a family and its members to chronic conditions in children and can be used to better understand the family’s efforts to incorporate caregiving demands (e.g., coordinating intervention services, managing challenging behaviors) into daily family life (Knafl & Deatrick, 2003; Knafl et al., 2012). In addition, the Family Management Measure (FaMM), is available as a quantitative measure of the dimensions of the FMSF (Knafl et al., 2009, 2011). Family management styles are patterns of response that are defined by how caregivers view the child, the condition, and the treatment regimen, their approach to managing the condition(s), and their perception of how the condition impacts family life now and into the future (Knafl et al., 2012). Research on children with chronic physical conditions identified four family management styles that range from Condition -Focused (i.e., family life is focused on the demands of managing the child’s condition) to Family -Focused (i.e., family life is centered on usual family routines; Knafl et al., 2013). Family-Focused management is associated with better family and child outcomes (e.g., improved family functioning, fewer child behavior problems, higher quality of life) when compared to families who were Condition-Focused (Knafl et al., 2013). These same management styles were also found in families of children with cancer (Deatrick et al., 2018), but patterns of family management have not yet been fully explored in ASD.

Nurses across specialty areas care for children with ASD and are well positioned to partner with families to provide care coordination and family-focused support (Giarelli & Gardner, 2012), However, nurses and other health care providers often report feeling unprepared to meet the child’s complex needs, thus indicating the need for additional resources to guide care (Mahoney et al., 2021; Walsh et al., 2020). The identification of patterns of family management, or family management styles, is a tool to aid nurses in identifying families at risk for poor outcomes and shows promise for nursing intervention development (Deatrick et al., 2018, 2022; Knafl et al., 2013). We focused this study on the caregiver’s assessment of their ability (Condition Management Ability, hereafter Ability) and their perceptions of the effort required (Condition Management Effort, hereafter Effort) to manage the child’s chronic condition(s) within the context of their daily family life. Ability and Effort were selected because increasing confidence in Ability and decreasing perceptions of Effort are potential modifiable targets for nursing interventions; however, we must first understand the modifiable factors that influence Ability and Effort.

### Child Factors

There is evidence from comparison of family management across chronic conditions, that characteristics of the child’s condition may influence family management (Kim et al., 2016; Van Riper et al., 2018). Specific to ASD, Kim et al. (2016) probed the influence of child behavior problems on family management assessed by the FaMM. When there were increased child behavior problems mothers reported that: (a) daily life was less normal, (b) they had lower confidence in their management ability, (c) management effort was higher, (d) there were greater difficulties with family life and (e) they had a more negative outlook on the child and family’s future (Kim et al., 2016). In that study, child behavior problems were inclusive of a diverse array of behaviors (aggression, self-injury, anxious behaviors, regulatory problems related to sleep or eating, and noncompliance) which precluded the examination of how distinct behaviors affect family management.

Feeding difficulties, sleep disturbances, and gastrointestinal symptoms are commonly occurring comorbid conditions in children with ASD (McElhanon et al., 2014; Sharp et al., 2013; Souders et al., 2017) and can have a particularly profound effect on families because they interfere with family routines like mealtime and bedtime. For example, Marquenie et al. (2011) found that dinnertime with children with ASD was often unpredictable, chaotic, and lacked meaningful family interactions which in turn increased familial stress and decreased cohesion. In contrast, Curtiss and Ebata (2019) using interviews & video recordings of mealtimes concluded that mealtimes are stressful, but parents do often successfully negotiate the experience. Having multiple co-occurring conditions may compound the impact on family life. For example, Prosperi et al. (2017), in a study of young children with ASD, found that food selectivity, sleep problems, and constipation clustered together; these children also had greater anxiety and self-injurious behavior, which can make family routines more challenging. Examining the relationships between child behaviors, co-occurring conditions, and family management can enhance our understanding of how some families successfully navigate family routines, while others experience challenges.

### Caregiver Mental Health

Caregivers of children with ASD often experience high stress, impaired parenting self-efficacy, and poor physical and mental health (Cohrs & Leslie, 2017; Ingersoll et al., 2011; Karst & Van Hecke, 2012). Depression, in particular, is significantly higher when the child has ASD (Cohrs & Leslie, 2017; Jackson et al., 2024). Maternal depressive symptoms as an influence on family management were examined by McKechnie et al. (2018) among mothers of young children (32–49 months) with developmental delay. Mothers with depressive symptoms tended to maintain a greater focus on negative child behaviors and developmental delays and viewed their own management ability more poorly.

### Family Social Support and Resources

In the FMSF, social support and resources are contextual influences on family management; however, relative to other aspects of the FMSF, contextual influences are understudied (Knafl et al., 2012, 2021). Caregivers of children with ASD may experience social isolation secondary to the unpredictability and intensity of their child’s behavior (Kirkpatrick et al., 2019; Myers et al., 2009). Importantly, social support can buffer stress related to having caregiving challenges and is associated with improved mental health and parenting self-efficacy (Ault et al., 2021; K. N. Hayes et al., 2023; Weiss et al., 2013). In addition to social support, adequate material resources (e.g., food security, housing stability) may ease family management. For example, a recent study by Stephenson et al. (2023) found that having strained resources was associated with lower parenting self-efficacy and greater parenting stress, factors related to family management.

### Study Aim

Using the FSMF as the theoretical framework, the overall aim of this study was to examine patterns of family management among families of children with ASD. The first sub aim was to examine the intersection of Ability and Effort, quantitatively measured by the FaMM and qualitatively explored in semi-structured interviews. The second sub aim was to examine the influence of child (ASD-related behaviors, feeding difficulties, sleep disturbances, gastrointestinal symptoms, aggression, self-injury), caregiver (anxiety, depression), and family (social support, unmet social needs) factors on family management pattern using both quantitative measures and qualitative interview data. This research can provide a roadmap for designing nursing care not only for children with ASD but also their families.

## Method

### Study Design

We conducted a convergent parallel mixed methods (QUAN + qual) study, in which quantitative and qualitative data were collected in parallel, analyzed separately, and then integrated in the final step (Creswell & Plano Clark, 2018). The purpose of using a convergent parallel mixed methods design for this study was to directly compare and contrast the quantitatively defined family management patterns with qualitative descriptions of how caregivers manage caregiving challenges (Creswell & Plano Clark, 2018). The quantitative strand (QUAN) was given priority because during data integration the patterns of family management were defined by the quantitative results on the FaMM Ability and Effort scales. In addition, in the QUAN (Page et al., 2024), we also compared the six dimensions of family management between families of children with ASD and families of children with Down Syndrome. The quantitative, qualitative, and integrated results reported in this paper include only caregivers of children with ASD. Institutional Review Board approval was received from the university and hospital involved in this study.

### Inclusion and Exclusion Criteria

Participants were the primary caregivers of children with ASD aged 4–10 years, inclusive. Primary caregiver was defined as an adult (≥18y) who lives with and self-reports that they take primary responsibility for the day-to-day care of the child. Only one primary caregiver per family participated in the study. The child’s ASD diagnosis was confirmed by electronic health record (HER) documentation, which could include scores on the Autism Diagnostic Observations Scale-2 (ADOS-2; Lord, Luyster, et al., 2012; Lord, Rutter, et al., 2012) or Childhood Autism Rating Scale-2, CARS-2 (Schopler et al., 2010), or clinician report of *Diagnostic and Statistical Manual of Mental Disorders* (5th ed.; *DSM-5*; American Psychiatric Association, 2013) criteria. Additional inclusion criteria included access to a computer/tablet device with internet and ability to complete study activities in English. Exclusion criteria included the child having a comorbid diagnosis of Down Syndrome because caregivers of children with Down Syndrome were recruited as a comparison group for the QUAN (Page et al., 2024). Caregivers of children fed by enteral tube were also excluded because the caregiving responsibilities for children fed by enteral tube differ significantly from those who are fed by mouth.

### Recruitment and Enrollment

We recruited a convenience sample of primary caregivers of children with ASD from an urban children’s hospital with an autism center of excellence. Children with ASD aged 4–10 years were identified through the hospital’s HER and their parent/guardian was emailed a flyer with information about participating in the study. After completing a screening questionnaire on REDCap, eligible participants provided written consent and were emailed a secure link to complete the quantitative study instruments (described below). All eligible participants who consented to participate were enrolled the QUAN of the study.

Enrollment in the qualitative strand occurred in parallel and was comprised of a nested sample of 30 caregivers selected from those who completed the QUAN. Maximum variation sampling (Creswell & Plano Clark, 2018) was used to select this nested sample. Specifically, using data collected during eligibility screening, we selected participants who were diverse in terms of: caregiving role (e.g., mother, father, grandparent), race/ethnicity, child’s age, and child’s gender. By using maximum variation sampling we aimed to gather a range of perspectives from caregivers of different backgrounds and with diverse family experiences. Having a diversity of perspectives helped us to identify shared and different experiences across the patterns of family management. A sample size of 30 allowed for an in-depth qualitative exploration of family management and using a nested sample allowed for direct comparison of the qualitative and quantitative data for these 30 participants (Creswell & Plano Clark, 2018).

Data collection in the quantitative and qualitative strands of the study occurred concurrently between September 2022 and February 2023. Qualitative interviews were scheduled within two weeks of the participant completing the QUAN of the study. Remuneration for participation was via electronic gift cards and participants received $15 for completing the QUAN and $20 for completing the qualitative strand.

### Quantitative Data Collection

The quantitative data was comprised of caregiver-report measures that were administered via REDCap.

The FaMM (Knafl et al., 2011) was used to assess the dimensions of family management that are described by the FMSF. The respondent rates each item about how their family manages the child’s chronic condition(s) from strongly disagree to strongly agree. The focus of this study was on the Condition Management Ability and Condition Management Effort scales. The Condition Management Ability scale assesses the caregiver’s perceptions of their knowledge about what needs to be done to care for the child’s condition(s) and their ability to competently carry out management responsibilities. Scores on the Condition Management Ability scale range from 12 to 60 with higher scores indicating that the caregiver perceives that the condition as more readily manageable. The Condition Management Effort scale assesses the caregiver’s perceptions of the time and work that is needed to manage the condition(s). Scores on the Condition Management Effort scale range from 4 to 20 with higher scores indicating that the caregiver views the condition(s) as requiring more effort to manage. All scales have good internal consistency (Kim et al., 2016; Knafl et al., 2011). In this sample, Cronbach’s alpha were .81 and .74 on the Condition Management Ability and Condition Management Effort scales, respectively. Reliability data on the other scales of the FaMM in this study can be found in Page et al. (2024).

Child (ASD-related behaviors, feeding difficulties, sleep disturbances, gastrointestinal symptoms, aggression, self-injury), caregiver (anxiety, depression), and family (social support, unmet social needs) factors that may influence family management were assessed quantitatively as described below.

The *Social Responsiveness Scale-2 (SRS-2)* assesses the child’s social awareness, social cognition, social communication, social motivation, and autistic mannerisms over the last 6 months (Constantino, 2012). Higher scores indicate greater ASD symptomatology. The SRS-2 has excellent internal consistency (Cronbach’s alpha >.95; Constantino, 2012). Excellent internal consistency was also found in this study (Cronbach’s alpha = .95).

The *Behavioral Pediatrics Feeding Assessment Scale (BPFAS)* measures child feeding in terms of dysphagia, selective intake, and food refusal (Crist & Napier-Phillips, 2001). Across chronic conditions, the BPFAS has demonstrated adequate internal consistency (Cronbach’s alpha >.76) and construct validity was assessed specifically in ASD (Allen et al., 2015; Crist & Napier-Phillips, 2001). The reliability in this sample was good, with a Cronbach’s alpha = .85.

The *Children’s Sleep Habits Questionnaire (CSHQ)* assesses eight types of sleep disturbances: bedtime resistance, sleep onset delay, sleep duration, sleep anxiety, night wakings, parasomnias, sleep-disordered breathing, and daytime sleepiness (Owens et al., 2000). Prior research using this scale with children with ASD found that reliability is acceptable (Cronbach’s alpha = .68; Johnson et al., 2012). Cronbach’s alpha was higher in this sample at .86.

The *Autism Speaks-Autism Treatment Network (AS-ATN) GI Signs and Symptoms Inventory-17 (GISSI-17)* screens for GI symptoms over the last 3 months with response options of “yes,” “no” and “unsure” (Margolis et al., 2019). Scoring of the inventory identifies children (screen positive or screen negative) with symptoms of constipation, diarrhea, and/or GERD. This scale does not require that the child be able to communicate or localize pain to their caregiver. Validation of the inventory is ongoing (Margolis et al., 2019).

The *Patient Health Questionnaire-9 (PHQ-9)* and the *Generalized Anxiety Disorder-7 (GAD-7)* were used to assess the caregiver’s feelings of depression and anxiety, respectively (Kroenke et al., 2001; Spitzer et al., 1994, 2006). The total scores can be used to classify severity of depression or anxiety symptoms. Internal consistencies are excellent (Kroenke et al., 2001; Spitzer et al., 2006). Reliability of each scale was good with Cronbach’s alphas of .87 and .89 for the PHQ-9 and GAD-7, respectively.

The *Accountable Health Communities Health Related Social Needs (AHC-HRSN Screening Tool* assesses unmet, non-medical social needs that can potentially impact health and access to health care (Billioux et al., 2017). Responses that indicated housing instability, food insecurity, lack of transportation to medical appointments, or unmet utility needs (i.e., threat or actual loss of electricity, gas, oil, or water services) were dichotomized to identify families with and without unmet social needs.

The family’s social support was evaluated with the *Social Support Index* (SSI; McCubbin et al., 1996). Higher scores indicate that the respondent feels more support from family, friends, and their community. Internal reliability in a sample of children with ASD (Cronbach’s alpha = .84; Hall, 2012) is good. Cronbach’s alpha (.93) was high in the current study.

### Qualitative Data Collection

The first author led qualitative data collection. She has prior experience with qualitative interviewing, and received additional training from the senior author, a qualitative expert. First, a theoretically based semi-structured interview guide was developed to reflect the dimensions of the FMSF and expand upon items on the FaMM. Then, pilot interviews with three caregivers (not included in this study) were conducted by the first author and then reviewed by the second author, and senior author. The research team then reviewed these pilot interviews and minor revisions to the interview guide were made to improve clarity of questions and ensure coverage of topics. The full interview guide is available in Supplemental Material 1. We first asked questions about management of the child’s overall health and behavior and then explored management of mealtimes and bedtimes, specifically. For example, an item on the FaMM Condition Management Ability scale is “We often feel unsure about what to do to take care of our child’s condition.” In the qualitative interview we further explored this by asking the participant to tell a story about a challenging mealtime/bedtime and describe how they decided what to do in those challenging situations. To elicit descriptions of Condition Management Effort, we asked caregivers to identify the areas where their child needs the most support and then describe how they help them with those support needs. The contextual influences on Ability and Effort were explored with questions like, “What kinds of things influence how you feel about your ability to help your child with . . .?” or “How do you decide what to do when . . .?” Quantitative measures were not scored prior to interviews, however, the BPFAS (feeding) and CSHQ (sleep) were reviewed so that the interview questions could be adapted to explore the child’s specific feeding or sleep challenges (e.g., “On the feeding questionnaire, you noted that you get frustrated and/or anxious when feeding your child, can you tell me more about that?”). In addition to pre-determined interview questions, probes such as “tell me more about that” or “please give me an example of. . .” were used to gather additional information from the participant responses. The exact order and phrasing of questions was flexible to facilitate a conversational interview between the researcher and participant. Further, the use of open-ended questions and probes encouraged participants to respond in detail, creating thick descriptions that aided in establishing transferability, or the extent to which the findings have applicability in other contexts (Lincoln & Guba, 1985).

The interviews, conducted by the first author via Zoom videoconference, were electronically recorded and field notes were taken after the interviews. Biweekly meetings between the first author and senior author were used to monitor fidelity of the interview technique and to explore otherwise implicit aspects of the inquiry process, reflect on interpretations, and discuss emerging findings which strengthens credibility (Lincoln & Guba, 1985). Electronic recordings were transcribed verbatim by the first author (nine interviews) or a professional transcription service (21 interviews). The first author then reviewed all written transcripts against the original audio file to ensure transcription accuracy and full de-identification prior to coding. The data management software ATLAS.ti (Version 22) was used to store and organize the qualitative data.

### Quantitative Data Analysis

IBM SPSS Statistics (Version 29) was used to analyze the quantitative data. Descriptive and graphical approaches were used to describe the quantitative data. Means and standard deviations are reported for continuous data and frequency distributions are presented for categorical data. Missing data were sporadic but did impact calculation of the total scores for several scales. To avoid underestimating these scores, we addressed missing values as follows. For the FaMM, we followed the scoring procedures to calculate scale scores with missing items (Knafl et al., 2009). For the SRS-2, we imputed the median value for each missing item, as detailed in the instrument manual (Constantino, 2012). For all other scales, we imputed the mean of the remaining items on the scale.

The distribution of each variable was assessed by inspecting histograms and using the Kolmogorov-Smirnov test. Owing to the small sample size and non-normality of some variables, non-parametric tests were used. To explore this sample more fully in terms of intra-group variation, the sample was split based on the median values for Condition Management Ability (hereafter, Ability) and Condition Management Effort (hereafter, Effort). This created four groups: (a) Ability ≥43, Effort <14; (b) Ability ≥43, Effort ≥14; (c) Ability <43, Effort <14; (d) Ability <43, Effort ≥14. Categorical variables (child gender, caregiver gender, constipation, diarrhea, GERD, aggression, self-injury, unmet social needs) were compared between groups using Fisher-Freeman-Halton tests. Kruskal–Wallis tests were used to evaluate differences in continuous child (age, feeding difficulties, sleep disturbances, ASD-related behaviors), caregiver (depression, anxiety), and family (social support) variables between these four groups. Post hoc tests to evaluate differences between pairs of groups were completed using the Dunn-Bonferroni approach that adjusts for multiple comparisons.

### Qualitative Data Analysis

Directed content analysis was used to theoretically integrate the dimensions of the FMSF with the experiences described in the interviews (Graneheim & Lundman, 2004; Hsieh & Shannon, 2005; Thompson et al., 2022). A priori codes were developed by the first author, second author, and senior author based on the FMSF, the six scales of the FaMM, and the pilot interviews. These codes were further revised, and new codes added based on the content of the interviews. For example, an a priori code of “Condition Management Effort,” a scale on the FaMM, was used to broadly code text related to the caregiver’s assessment of the time and effort needed to manage the child’s condition during the first read of the transcripts. Additional codes related to routines, planning, and types of intervention services were added during iterative review of the transcripts. A codebook was used to document the codes and definitions. An audit trail of coding decisions was maintained, and memos were written to capture thoughts and impressions during analysis (Hsieh & Shannon, 2005). Finally, themes and subthemes were developed to link meaning across the codes (Graneheim & Lundman, 2004).

The first four interviews were coded by the first author and senior author to establish coding agreement. The remaining 26 interviews were coded by the first author. To address limitations of a single coder, a random sample of 9 interviews (35% of sample) were reviewed by the second author to evaluate that codes were consistently applied per the codebook definitions. In addition, the first author met regularly with the senior author and the third author to reflect on the interviews and analysis process in the context of the researchers’ prior clinical experience, values, and cultural perspectives. The audit trail and reflexive techniques served to establish confirmability (Lincoln & Guba, 1985). Finally, during regularly scheduled meetings, the study team met to review the findings to ensure that the data supported the interpretation, a process that supports dependability (Lincoln & Guba, 1985).

### Integrated Data Analysis

The intent of integrating data in this convergent parallel mixed methods study was to expand understanding of how families of children with ASD manage caregiving challenges. The conceptual anchors in this mixed methods study were two aspects of family management, Ability and Effort. Each case was comprised of the quantitative and qualitative data collected from a primary caregiver about the child, the caregiver, and the family unit. A joint display comparing the qualitative themes and quantitative data across the 30 cases was created to facilitate data integration. This joint display was used to determine the ways in which the results from qualitative strand and QUAN of the study confirm, disconfirm, or expand each other (Creswell & Plano Clark, 2018). Specifically, the qualitative themes and subthemes that correspond to the concepts of Ability and Effort in the FMSF were identified and described within each case. Then, the cases were sorted into the four groups defined by their quantitative scores on the FaMM Ability and Effort scales. Qualitative themes and subthemes were compared across cases and within each group to evaluate similarities and differences between groups.

## Results

### Sample Characteristics

Sample demographics and clinical characteristics are summarized in Tables 1 and 2. In the QUAN (*n*=56), caregivers were 91.1% Female, 76.8% White, and had a mean age of 38.43 (SD = 6.72). Most had a college (37.5%) or graduate degree (39.3%) and the majority were working full (51.8%) or part-time (23.2%). The children were predominantly male (71.4%) with a mean age of 6.71 (SD = 2.23). In addition to ASD, the most common co-occurring diagnoses reported by parents were anxiety (44.6%), aggressive behavior (37.5%), attention deficit hyperactivity disorder (37.5%), allergies (33.9%), sleep problems (32.1%) and feeding/eating problems (28.6%). About one-third (30%) were on a prescription medication for behavioral or psychiatric symptoms. In terms of services, 85.7% had an individualized education plan in school, and nearly all children received at least one therapy in school (94.6%). The majority (55.4%) received at least one outpatient therapy, and about half of the sample (51.8%) received therapy services both in-school and as outpatients.

**Table 1.** Participant Demographics.

Variable	Quantitative sample (*n* = 56)	Nested qualitative sample (*n* = 30)
Caregiver age	38.43 ± 6.72	38.67 ± 7.06
Caregiver’s gender		
Female	51 (91.1%)	27 (90%)
Male	5 (8.9%)	3 (10%)
Child’s age	6.96 ± 1.82	6.90 ± 1.69
Child’s gender		
Female	16 (28.6%)	10 (33.3%)
Male	40 (71.4%)	20 (66.7%)
Caregiver ethnicity		
Hispanic	7 (12.5%)	4 (13.3%)
Non-Hispanic	49 (87.5)	26 (86.7%)
Caregiver Race^a^		
American Indian or Alaska Native	1 (1.8%)	1 (3.3%)
Asian	5 (8.9%)	4 (13.3%)
Black or African American	7 (12.5%)	4 (13.3%)
Native Hawaiian or Other Pacific Islander	1 (1.8%)	1 (3.3%)
White	43 (76.8%)	22 (73.3%)
Prefer not to answer	2 (3.6%)	1 (3.3%)
Child ethnicity		
Hispanic	10 (17.9%)	6 (20%)
Non-Hispanic	46 (82.1%)	24 (80%)
Child Race^a^		
American Indian or Alaska Native	1 (1.8%)	1 (3.3%)
Asian	5 (8.9%)	4 (13.3%)
Black or African American	10 (17.9%)	5 (16.7%)
Native Hawaiian or Other Pacific Islander	1 (1.8%)	1 (3.3%)
White	44 (78.6%)	24 (80%)
Prefer not to answer	2(3.6%)	1 (3.3%)
Relationship status		
Single	8 (14.3%)	3 (10%)
Living with partner	48 (85.7%)	27 (90%)
Education		
High school diploma	10 (17.9%)	8 (26.7%)
College degree	21 (37.5%)	12 (40%)
Graduate degree	22 (39.3%)	8 (26.7%)
Other	3 (5.4%)	2 (6.7%)
Employment		
Student or Trainee	3 (5.4%)	2 (6.7%)
Working Part Time (less than 35 hours/week)	13 (23.2%)	5 (16.7%)
Working Full Time (35 hours/week or more)	29 (51.8%)	16 (53.3%)
Unemployed	3 (5.4%)	2 (6.7%)
Unable to work due to illness or disability	4 (7.1%)	3 (10%)
Unable to work due to child’s illness or disability	2 (3.6%)	2 (6.7%)
Other	2 (3.6%)	0
Finances	n=55	n=29
Have more than enough to make ends meet	16 (28.6%)	6 (20.7%)
Have enough to make ends meet	28 (50%)	16 (55.2%)
Do not have enough to make ends meet	11 (19.6%)	7 (24.1%)

*Note*. Mean ± SD are provided for continuous variables. *N*(%) are provided for categorical variables.

a Sum of responses exceeds sample because participants could select multiple race categories.

**Table 2.** Caregiver Report of Child’s Health, Behavior, and Services.

Variable	Quantitative sample (*n* = 56)	Nested qualitative sample (*n* = 30)
Caregiver report of child’s health & behavior		
Allergies	19 (33.9%)	10 (33.3%)
Aggressive behavior	21 (37.5%)	10 (33.3%)
Anxiety	25 (44.6%)	14 (46.7%)
Asthma	9 (16.1%)	6 (20%)
Attention deficit hyperactivity disorder	21 (37.5%)	10 (33.3%)
Epilepsy/seizure disorder	3 (5.4%)	3 (10%)
Heart condition	3 (5.4%)	1 (3.3%)
Feeding or eating problems	16 (28.6%)	9 (30%)
Genetic or inherited condition	7 (12.5%)	3 (10%)
Self-injurious behavior	9 (16.1%)	5 (16.7%)
Sleep problems	18 (32.1%)	9 (30%)
School services		
Individualized Education Plan (IEP)	48 (85.7%)	26 (86.7%)
504 plan	4 (7.1%)	2 (6.7%)
Applied behavior analysis	21 (37.5%)	10 (33.3%)
Counseling	10 (17.9%)	6 (20%)
Physical therapy	15 (26.8%)	6 (20%)
Occupational therapy	38 (67.9%)	19 (63.3%)
Social skills group	29 (51.8%)	17 (56.7%)
Speech therapy	42 (75%)	20 (66.7%)
Outpatient therapy services		
Applied behavior analysis	14 (25%)	8 (26.7%)
Counseling	8 (14.3%)	5 (16.7%)
Occupational therapy	11 (19.6%)	7 (23.3%)
Physical therapy	2 (3.6%)	2(6.7%)
Social skills group	3 (5.4%)	2 (6.7%)
Speech therapy	8 (14.3%)	5 (16.7%)

After completing the quantitative instruments, 50 participants (89.3%) agreed to be contacted and 30 participants completed the qualitative interview. Interviews averaged 43.3 minutes in length (range: 22.8 minutes to 70.1 minutes).

### Quantitative Results

On the FaMM, the mean of Ability was 42.39 (SD = 7.54) and the mean of Effort was 13.64 (SD = 3.79). The majority of the sample were in the groups with lower ability, higher effort (37.5%) or higher ability, lower effort (35.7%). In addition, 17.9% of cases were in the higher ability, higher effort group and 8.9% of cases were in the lower ability, lower effort group. The Kruskal–Wallis tests showed significant differences between the four groups for scores on the BPFAS (*H* (3) = 7.911, *p* = .048), CSHQ (*H* (3) = 8.948, *p* = .03), SRS-2 (*H* (3) = 22.241, *p* < .001), and SSI (*H* (3) = 10.413, *p* = .015). The post hoc analysis identified statistically significant differences between the higher ability, lower effort group and the lower ability, higher effort group with respect to CSHQ (*p* = .019), SRS-2 (*p* < .001), and SSI scores (*p* = .009). Group differences with respect to the BPFAS score were no longer significant in the adjusted analysis. There were also significant differences in aggression and self-injury using Fisher-Freeman-Halton Exact tests (see Table 3).

**Table 3.** Group Comparison of Child, Caregiver, and Family Factors.

Variable	Quantitative sample	Higher ability, lower effort	Higher ability, higher effort	Lower ability, lower effort	Lower ability, higher effort	Fisher-Freeman-Halton Test, *p*-value	Kruskal Wallis Test, *p*-value
*n*	56	20	10	5	21		
Demographic factors							
Child’s age	6.96 ± 1.82	7.15 ± 2.06	7.7 ± 1.64	5.6 ± 1.52	6.76 ± 1.61		.174
Child’s gender (% male)	40 (71.4%)	16 (80%)	6 (60%)	3 (60%)	15 (71.4%)	.622	
Caregiver’s age	38.43 ± 6.72	38.3 ± 6.77	37.2 ± 6	35.2 ± 6.69	39.90 ± 7.07		.429
Caregiver gender (% female)	51 (91.1%)	19 (95%)	9 (90%)	4 (80%)	19 (90.5%)	.634	
Child factors							
BPFAS Child Behavior Frequency Score	56.93 ± 14.02	51.25 ± 10.91	62.5 ± 14.97	51.8 ± 17.82	60.9 ± 13.86		.048*
CSHQ total	50.00 ± 9.85	45.85 ± 9.16	50.50 ± 8.26	48 ± 10.65	54.19 ± 9.85		.03*
SRS-2 Total Raw Score	96.59 ± 30.90	76.65 ± 25.59	88.60 ± 25.52	95 ± 25.52	119.76 ± 24.22		<.001***
GISSI-17: Constipation (Y/N)	36 (64.3%)	11 (55%)	8 (80%)	3 (60%)	14(66.7%)	.595	
GISSI-17:Diarrhea (Y/N)	35 (62.5%)	10 (50%)	7 (70%)	4 (80%)	14(66.7%)	.559	
GISSI-17: GERD (Y/N)	21 (37.5%)	7 (35%)	5 (50%)	0	9 (43%)	.294	
Aggression (Y/N)	21 (37.5%)	6 (30%)	7 (70%)	0	8 (38.1%)	.048*	
Self-injury (Y/N)	9 (16%)	0	4 (40%)	0	5 (23.8%)	.005**	
Caregiver factors							
PHQ-9 Total Score	6.61 ± 5.53	4.70 ± 3.59	9.6 ± 7.74	5.2 ± 5.36	7.38 ± 5.51		.218
GAD-7 Total Score	7.27 ± 5.08	6.05 ± 3.36	9.20 ± 6.41	6.4 ± 5.94	7.71 ± 5.56		.646
Family factors							
SSI total Score	46.13 ± 12.31	52.9 ± 8.06	45.8 ± 11.99	43.6 ± 9.97	40.42 ± 13.74		.015*
≥1 Unmet social need	17 (30.4%)	6 (30%)	4 (40%)	3 (60%)	4 (19%)	.267	

* *p* < .05. **p < .01. ***p < .001.

### Qualitative Results

The qualitative analysis highlighted the complex factors that influence how caregivers perceive their own ability to manage ASD and co-occurring conditions as well as the effort that is required for management. Each theme is briefly described below. Tables 4 and 5 were constructed from the transcript files, code book and analytic memos, to demonstrate how the codes were used to develop the themes and subthemes.

**Table 4.** Qualitative Theme #1: Developing Management Ability Is a Dynamic Process of Involving Others, Learning From Experience, Managing Uncertainty, and Negotiating Competing Priorities.

Subtheme	Subtheme description	Codes	Code definition	Exemplar quote
Receiving support from others	Caregivers identified various sources of knowledge and support, including family members, therapists, teachers, healthcare providers, internet research, and other parents. However, the quality and quantity of support received from others impacted perceptions of Ability.	Family support	Support from family members	We have a very close-knit family with my immediate family. My mom. My dad. Um, very involved—like, overly involved (Participant 19)
		Community support	Support from the community (including therapists, teachers, healthcare providers, other parents)	I can’t say whether or not that’s good for her ’cause I’m not an educator. . .so I just really had to talk with her therapist, and they were like, ‘oh, we think that that’s not gonna be the best for her’ (Participant 13) ‬‬‬‬‬‬‬‬‬‬‬‬‬
		Virtual support	Support from internet resources	There’s autism support groups on, like, Facebook, and you can ask, like, ‘Hey, you know, my child’s having a really hard time in the bath’. . . people give, you know, ‘Hey, this is what I do.’(Participant 10)
		Isolation/stigma	Feeling isolation or stigma from family or community	We haven’t told, like, a lot of family members because I feel like people will treat her differently because of that, you know? (Participant 14)
Learning from experience	Caregivers described using “trial and error” to try different behavior management strategies and find what worked for their child. They also relied on experience parenting other children.	Condition management experience	How caregivers use past experience to shape decisions of management behaviors	It was trial and error, like, just figuring out what worked for him (Participant 4)
		Parenting experience	How caregivers use experience parenting siblings.	I’ve learned with [older sibling] that a solid bedtime helps with getting everybody to sleep. ‬‬‬‬‬‬‬(Participant 14)
Managing uncertainty	Managing uncertainty was integral to the process of developing Ability as all caregivers described having feelings of uncertainty about “what to do” and experienced times where their questioned their own management ability	Management Uncertainty	Feeling uncertain or questioning management ability	Did I handle that situation the best that I could have handled? Is that going to affect him in the long run? (Participant 21)
		Caregiver Worry	Feeling worried about the child’s condition	I wonder and worry are we doing the right thing? Are we going to the right provider? What is it, you know, we should do? (Participant 8)
Negotiating competing priorities	When deciding how to manage a specific behavior (e.g., food refusal, bedtime resistance), caregivers described having to negotiate competing priorities	Competing Priorities	Descriptions of two or more priorities in conflict	That’s [co-sleeping] somethin’ I would love to change, but, at the same time, I want her to just get the sleep that she needs. And I’m not gonna lie. In all honesty, when she falls asleep, and I just have that quiet time, whether it’s if I’m stuck in the bed or anywhere else if I just have that quiet time before I go to sleep, I’m happy with it. (Participant 29)
		Management Decision-Making	Caregiver’s descriptions of how they decide what to do about their child’s behavior or medical condition.	This is where is gets a little tricky right, like, what is his mood like? Because if he’s just asking for something out of habit, I can ask him to eat something healthier before I give him his preferred item (Participant 2)

**Table 5.** Qualitative Theme #2: Effort Involves Managing Routines, Thinking Ahead, and Coordinating Services.

Subthemes	Subtheme description	Codes	Code definition	Exemplar quote
Managing routines	Caregivers described utilizing routines to support their child through the day. However, while some routines centered on the child’s needs (child-centered routines) others incorporated the needs of all family members (family-centered routines).	Child-centered routines	Routines that are centered on the child’s needs	we do everything we can to like, keep him and his schedule running smoothly and like plan out transitions, so that we minimize his risk for having, like an epic meltdown (Participant 2)
		Family-Centered Routines	Routines that incorporate the needs of all family members	I’ve tried to keep [bedtime] very routine and consistent, just because it makes my life easier. And I know there is an end in sight (Participant 23)
Thinking Ahead	Caregivers described the mental effort needed to plan ahead and prepare for potentially difficult situations. This included planning for both the short- and long-term.	Planning- Daily	Planning daily activities.	We’re constantly scheduling around him. And you know, thinking of things like that [taking 2 cars to an event] (Participant 16)
		Planning- Future	Planning for the future.	If I need to be, like, a power of attorney. . .he’s not at the place where he can make his own decisions. (Participant 18)
		View of condition impact in the future	The caregiver’s description of how serious the condition will be for the child in the future.	I feel like he’s always gonna kinda need that extra eye on him (Participant 21)
Coordinating Services	Caregiver’s efforts to support their child’s development included attending medical appointments, working with therapists, and coordinating school services. Effort was variable depending on the amount of supports the child required as well as the barriers caregivers faced to accessing services.	Medical Services	Use of medical services	It’s been about a year since we’ve seen a doctor because of, like the whole scheduling thing is very hard for me to manage. (Participant 7)
		Educational Services	Use of educational services	Trying to figure out the best approach for her education plan was just another layer to everything (Participant 13)
		Therapy Services	Use of therapy services	We have to take [her] to therapies. We have to take her to additional stuff. . .fun therapies like from music, swimming (Participant 5)

#### Theme 1: Developing Management Ability Is a Dynamic Process of Involving Others, Learning From Experience, Managing Uncertainty, and Negotiating Competing Priorities

During the interviews, caregivers described their management ability in terms of their knowledge about how to manage specific behaviors and reflected on how they feel about their own ability to manage day-to-day caregiving responsibilities and challenges. Caregivers described managing their child’s health and behavior as a constantly evolving process. The subthemes of “Receiving Support from Others,” ‘Learning from Experience “Managing Uncertainty” and “Negotiating Competing Priorities” describe how caregivers learned strategies, variously considered the needs of the child and the family, and validated their roles as parents and caregivers (see Table 4).

#### Theme 2: Effort Involves Managing Routines, Thinking Ahead, and Coordinating Services

Caregivers described that their efforts to manage their child’s health and behavior were time-consuming and reached across multiple domains. The subthemes of “Managing Routines,” ‘Planning Ahead,’ and “Coordinating Services” describe their work as parents and caregivers of children with ASD (see Table 5).

### Integrated Results

**Figure 1.**
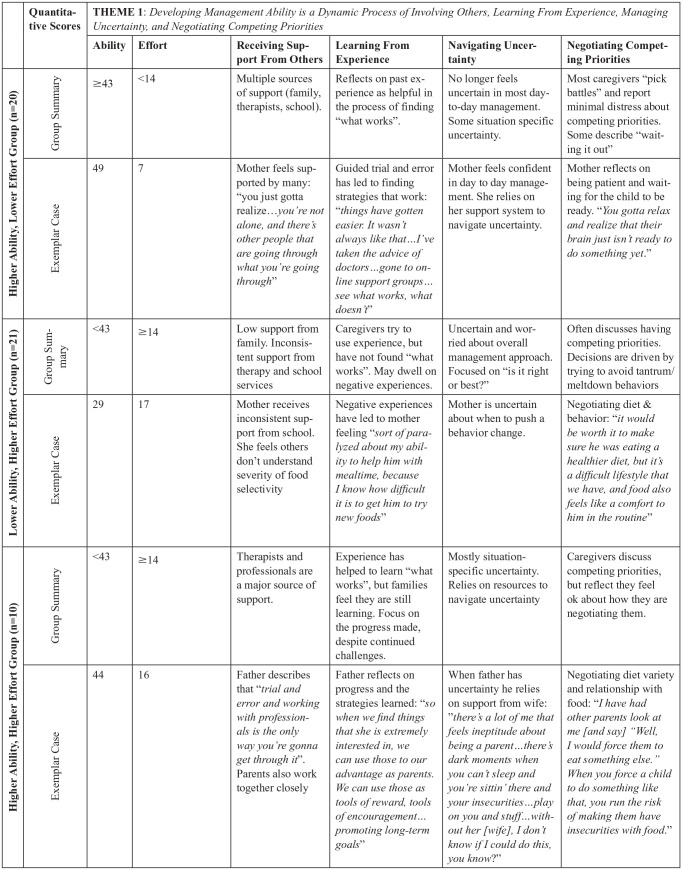
Example of Joint Display for Theme 1.

**Figure 2.**
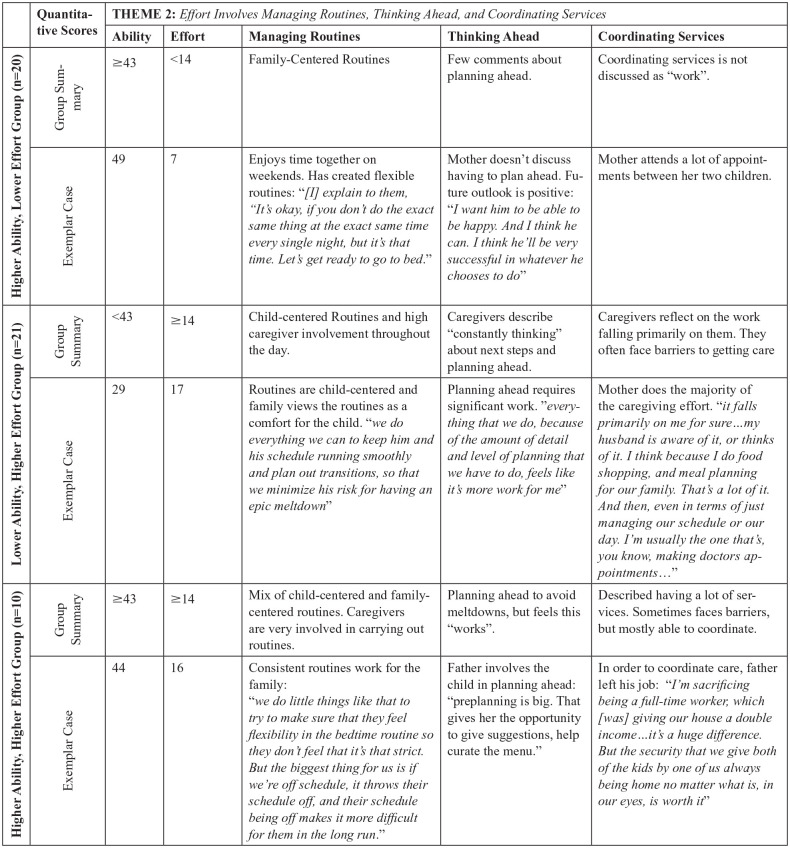
Example of Joint Display for Theme 2.

Integration of the quantitative and qualitative data revealed differences in the qualitative themes between groups. The caregivers’ qualitative descriptions of Ability existed on a continuum, ranging from unsure to confident, and converged with the quantitative scores on the FaMM Ability scale. Further, management ability was dynamic and changed over time as well as in response to changes in their child’s behavior or changes in the family. The degree to which Ability was responsive to the child’s behavior varied across groups. While all caregivers endorsed devoting significant time and resources to managing their child’s health and behavior, there was variability in how Effort was perceived. While some caregivers perceived the Effort as manageable and well-integrated into their daily family routines, others perceived the Effort as overwhelming. These qualitative differences help to explain the quantitative associations found between various child, caregiver, and family factors and condition management ability and effort. A sample of the joint displays are included in Figures 1 and 2 to demonstrate how the quantitative and qualitative data were integrated within case examples.

#### Higher Ability and Lower Effort

Caregivers in the group with higher ability and lower effort (FaMM scores: Ability ≥43, Effort <14) expressed confidence in their abilities and the work of day-to-day management was incorporated into family-centered routines that balanced the child’s needs with the needs of other family members.

Caregivers described receiving support from multiple sources (family, therapists, school) to learn strategies to manage behavior (e.g., tantrums) or help their child to learn a new skill (e.g., potty training). They recalled learning from past experience and viewed this experience as helpful and validating in figuring out “what works” for their family. Caregivers explained that condition management now was easier than in the past. Uncertainty expressed in this group tended to be about the past or situation-specific (e.g., uncertain about how to increase food variety). In addition, caregivers focused on improvements in their child’s behavior (e.g., no longer eloping, improved communication). This qualitatively described developmental progress converged with the quantitative result that ASD symptoms were less severe in this group compared to the lower ability, higher effort group (described below).

Caregivers in this group did still face challenges to managing their child’s health and behavior. As we probed mealtime and bedtime challenges in the interviews, concerns about bedtime were rare, but mealtime was often a challenge due to the child’s selective diet. As caregivers negotiated trying to introduce new foods, they often described “picking their battles” or feeling that they just needed to be patient and remained confident that they would most likely figure it out in the future.

#### Lower Ability, Higher Effort

The narrative accounts of caregivers in the lower ability, higher effort group (FaMM scores: Ability < 43, Effort ≥14) were markedly different. Caregivers in this group reported low confidence in their ability to manage their child’s health and behavior and described condition management as “exhausting” and all encompassing.

Caregivers in this group described receiving limited support from their extended family either due to geographic location or because family disagreed with or misunderstood the ASD diagnosis. Even when two partners lived in the home, the primary caregivers often described that the work of coordinating services and managing daily routines fell largely on them. Further, supports received from therapy providers and schools were inconsistent, thus creating barriers to care and additional work for the caregiver. These qualitative accounts confirm the quantitative finding that perceived social support was significantly lower in this group compared to the higher ability, lower effort group.

Caregivers in this group expressed overwhelming uncertainty about their overall management approach and frequently worried if they were “doing it right.” This uncertainty and worry seemed to stem from the fact that although they had tried multiple strategies (past experience) or had knowledge about “what to do” their child’s behavior continued to be unpredictable and often significantly disrupted family life (e.g., difficulty eating meals or attending events together).

When discussing specific challenges at mealtime and bedtime, food refusal, bedtime resistance, and nighttime wakings were frequently described. Caregivers’ decisions about how to approach these behaviors were largely driven by trying to avoid tantrums or meltdowns that were distressing for the both the child and family. However, caregivers in this group, unlike those in the higher ability, lower effort group, did not report feeling that they could “wait it out.” This is perhaps because these caregivers had seen less progress in their child’s behavior to date or the severity of the child’s feeding and/or sleep problems were greater. Indeed, scores on SRS-2 (autism symptoms) and CSHQ (sleep) were significantly higher in this group compared to the high ability, low effort group. Scores on the BPFAS (feeding) were higher, but not significantly so, in the adjusted analysis.

Daily routines were child-centered with significant effort dedicated to maintaining the child’s routine, even at the expense of the caregiver’s or family’s needs. Caregivers explained this was necessary because deviations could lead to the child experiencing distress or exhibiting behaviors that the family found more challenging to resolve (e.g., aggression). In addition, mealtimes, bedtimes, and other routines of daily living had high caregiver involvement, often beyond what would be expected for the child’s chronological age. Finally, caregivers often discussed that significant mental effort was dedicated to planning ahead and thinking about the child’s future.

#### Higher Ability, Higher Effort

In this group, caregivers had quantitatively higher ability and higher effort (FaMM scores: Ability ≥ 43, Effort ≥14). Although there were no statistically significant differences between child, caregiver, and family variables in this group and the three other groups, the qualitative data begin to uncover ways in which this group is unique.

Consistent with the higher effort scores on the FaMM, caregivers in this group described devoting significant effort to coordinating multiple types of services, managing daily routines, and thinking ahead to plan activities or anticipate changes to the routine. Carrying out mealtime and bedtime routines, specifically, required a lot of effort on the part of the caregiver. However, unlike the lower ability, higher effort group, caregivers in this group reported having a mix of family-centered and child-centered routines. That is, caregivers often felt able to balance their child’s higher caregiving needs with the family’s needs.

Despite high effort, caregivers described feeling confident in many areas of managing their child’s health and behavior. Focusing on past positive experiences and their child’s progress as well as relying on the support they received from others qualitatively differentiated this group from the lower ability, higher effort group. In addition, several caregivers spoke of rejecting perceived societal norms and pressures as a mechanism to improve confidence.

#### Lower Ability, Lower Effort

The fewest caregivers were in the lower ability, lower effort group (FaMM scores: Ability < 43, Effort < 14). Qualitative accounts tended to overlap with other groups, which could be attributed to most scores being borderline (e.g., effort =13, ability = 42) to a score that would change group assignment. One interesting finding in this group was that caregivers tended to describe their child as independent, an attribute that may confer lower effort for the caregiver. The characteristics of these children, caregivers, and families that make the condition less readily manageable despite this lower perceived effort, warrant further investigation.

## Discussion

This study examined patterns of family management among families of children with ASD and described the influence of child (ASD-related behaviors, feeding difficulties, sleep disturbances, gastrointestinal symptoms, aggression, self-injury), caregiver (anxiety, depression), and family (social support, unmet social needs) factors on family management pattern using both quantitative measures and qualitative interview data. The integrated analysis revealed that the severity of the child’s ASD behaviors and sleep problems as well as the caregiver’s perceived social support were associated with management effort and the extent to which caregiving responsibilities were integrated into daily life. When daily routines were child-centered (i.e., less integrated into daily life), caregivers expressed less confidence in their ability to manage daily challenges.

While a large body of research has defined family management styles among children with chronic conditions, this study is among the first to apply the FMSF in the population of families of children with ASD. Conceptually anchored in the FaMM measures of Condition Management Ability and Condition Management Effort, caregivers in three of the four quantitatively defined groups described qualitatively different approaches to family management. The most notable differences, both in terms of statistical associations and narrative accounts, were between the higher ability, lower effort and the lower ability, higher effort groups.

Prior work has identified four family management styles (Family-Focused, somewhat Family-Focused, somewhat Condition-Focused, Condition-Focused) among families of children with chronic physical conditions (Knafl et al., 2013) and adolescent and young adult brain tumor survivors (Deatrick et al., 2018). Relative to the other family management styles, a Family-Focused management style has the highest mean score in Condition Management Ability and the lowest mean score in Condition Management Effort whereas a Condition-Focused management style has the lowest mean score in Condition Management Ability and the highest mean score in Condition Management Effort. In the present study, caregivers in the higher ability, lower effort group described having daily routines that balanced supporting the child’s needs with those of the family, which is consistent with a Family-Focused management style. In contrast, caregivers in the lower ability, higher effort group described having daily routines that revolved around the child, with significant time and effort being devoted to carrying out care responsibilities and navigating recurrent challenges of condition management. These are characteristics of a Condition-Focused family management style. Thus, our higher ability, lower effort group aligned with the Family-Focused pattern and our lower ability, higher effort group aligned with the Condition-Focused pattern. Caregivers in the higher ability, higher effort group and lower ability, lower effort group were mixed in terms of the extent to which daily routines centered on the child’s health and behavioral needs. Similarly, Knafl et. al. (2013) and Deatrick et al (2018) found intermediary patterns of somewhat Family-Focused and somewhat Condition-Focused. Taken together, these findings suggest that the four previously validated family management styles may replicate in families of children with ASD; however, further study is needed in a larger sample.

In this sample, caregiver perceptions of the severity of their child’s ASD behaviors, sleep disturbances, and feeding difficulties were different between groups. Group comparisons indicated that caregivers in the lower ability, higher effort group rated their child’s ASD behaviors and sleep disturbances as significantly more severe than the higher ability, lower effort group. Feeding difficulties were not significantly different between groups after adjusting for multiple comparisons. These findings are similar to Kim et al. (2016) who found that increased child behavior problems, using a measure that included behaviors common in ASD (e.g., aggression, self-injury) as well as feeding and sleep concerns, were associated lower condition management ability and higher condition management effort on the FaMM. While it is logical that having more challenging behaviors, sleep disturbances, and feeding difficulties corresponds to greater caregiving demands and more complex care, the mixed methods nature of this study and the group comparisons allowed us to explore some of the nuance of these relationships.

The SRS-2 specifically quantifies the social differences that are diagnostic of ASD and higher SRS-2 scores are associated with lower adaptive functioning and more internalizing and externalizing behaviors (Hus et al., 2013; Tillmann et al., 2019). In addition, the presence of sleep disturbances and feeding difficulties have been linked to greater parent-reported behavioral concerns (Curtin et al., 2015; Mazurek et al., 2013, 2019; Postorino et al., 2015). The presence of aggression and self-injury may introduce particular challenges to family management as families navigate maintaining safety for the child and other family members. Indeed, this analysis indicated group differences in terms of the child’s aggressive and self-injurious behaviors. Specifically, the two groups with higher effort had a greater proportion of children with aggression and self-injurious behaviors. The narrative accounts of caregivers provided additional explanation of this result as caregivers described expending significant effort to maintain the child’s preferred routine and thus avoid situations where aggression and self-injury were more likely to occur. The qualitative data in this study also points to the unpredictability of behavior as being particularly challenging for families, a finding consistent with prior qualitative work (Marquenie et al., 2011; Ooi et al., 2016). Many caregivers in the lower ability, higher effort group described having knowledge about “what to do” and experience trying numerous approaches, but they had yet to find strategies that consistently worked to help their child through meltdowns, improve sleep, or increase food variety. These caregivers described overwhelming uncertainty and low confidence in their condition management ability.

Perceived social support by the primary caregiver was significantly higher in the higher ability, lower effort group compared to the lower ability, higher effort group. The qualitative data converged with this statistical association as caregivers in the higher ability, lower effort group described having support from their partner, extended family, and broader community. In contrast, caregivers in the lower ability, higher effort group more often described isolation from family as well as hesitance to participate in the community due to perceived judgment about their child’s behavior and/or their parenting ability. These results are in line with others who have found that higher perceived social support is linked to better caregiver mental health, lower stress, and improved family functioning (Ekas et al., 2010; K. N. Hayes et al., 2023; Robinson & Weiss, 2020; Whitehead, 2017). Supportive communities may also promote family resilience, even when the caregiver has poorer mental health (K. N. Hayes et al., 2023). Social support as a protective factor may help to explain why there were no differences between the four groups in terms of self-reported symptoms of depression and anxiety. Importantly, across the sample 26.8% and 30.3% of caregivers had clinically significant PHQ-9 and GAD-7 scores (>10), respectively.

Nurses across specialty areas will care for individuals with ASD and/or their caregivers. The results of this study point to two priority areas for nurses to support families in managing ASD: (a) shifting from child-centered to family-centered routines, and (b) identifying social supports. These recommendations can be incorporated into existing nursing practice, like the “15-minute Family Interview.” The “15-minute Family Interview” is a concise template for engaging with families and includes the essential components of manners, therapeutic conversation, family genogram, therapeutic questions, and commendations (Wright & Leahey, 1999). First, nurses can help caregivers to identify ways to shift mealtime, bedtime, and other daily routines from child-centered to family-centered. Using therapeutic conversation techniques like acknowledging the caregiver’s expertise in managing their own child’s behavior, nurses can inquire about routines that are “working well” for the family as well as routines that are currently challenging. Nurses can then talk through potential strategies for a targeted behavior (e.g., reduce bedtime resistance) and guide caregivers through the “trial and error” process of changing a routine. Helping families to identify routines that leverage the child’s strengths and are inclusive of the needs of all family members may help to reduce Effort and enhance Ability in this population. Second, the “15-minute Family Interview” includes family genograms and ecomaps as an essential component and these strategies can be used to assess caregivers’ social support networks. With an understanding of the caregivers’ current social supports, nurses can then help caregivers to identify new sources of support (e.g., a parent-to-parent support group in the community) or brainstorm ways that existing social supports can be bolstered to ease family management. Future research may consider testing an adapted “15-minute Family Interview” that is specific to ASD and includes these components.

The results of this study should be interpreted in light of several limitations. First, the total sample was predominately White mothers in two parent heterosexual couples. In addition, the majority of participants also had at least a college education and reported financial stability. The use of maximum variation sampling helped to ensure that the perspectives of fathers and non-White caregivers were included in the qualitative data; however, the generalizability of our findings is limited and future research should aim to recruit more fathers, caregivers from underrepresented ethnic and minority groups, and caregivers from different family structures. Our recruitment strategy that utilized the HER of an urban medical center with an autism center of excellence may have also biased the sample. These results may not be generalizable to families living in rural areas or families with less access to health care and autism-specific services. Second, all quantitative measures and interviews were completed by a single primary caregiver. Future research could compare perspectives of family management between multiple caregivers and, if developmentally appropriate, also elicit the perspective of the child with ASD and their sibling(s). In addition, the use of more objective assessments of ASD behaviors (e.g., clinician administered autism testing), sleep (e.g., actigraphy), and feeding (e.g., clinical evaluation) would strengthen the study. Third, a single coder was used for the majority of transcripts in the qualitative analysis. To minimize the potential bias of having a single coder, we used a second coder to review a random sample of transcripts and verify codes were applied according to their definitions. In addition, the study team met regularly to discuss the analysis and interpretation of findings. Finally, there were a small number of families in the lower ability, lower effort group, which limits the conclusions that can be drawn about this group. Using a sequential explanatory design in a future study with a large quantitative sample and then a purposeful qualitative sample would facilitate validation of the family management styles in ASD.

## Conclusion

This study demonstrated the applicability of the FMSF to families of children with ASD and identified four patterns of family management that are anchored in the concepts of Ability and Effort. While family management in the higher ability, lower effort group was characterized by care that was perceived as manageable and well-integrated into daily family life, caregivers in the lower ability, higher effort group expended considerable time and effort adhering to inflexible child-centered routines that impacted the lives and roles of other family members. A third pattern of family management (higher ability, higher effort) was differentiated by caregivers who were confident in their management ability despite high day-to-day management effort, a phenomenon supported by qualitative evidence that caregivers in this group had more family-centered than child-centered routines. The fourth pattern of family management (lower ability, lower effort) had few participants and requires further investigation. Finally, the data from the quantitative measures and qualitative interviews converged to identify that specific child characteristics (ASD behaviors, sleep disturbances, aggression, self-injury) as well as the caregiver’s perceived social support influenced how families of children with ASD approached management the child’s health and behavior. Nurses caring for children with ASD can support families in shifting daily routines from child-centered to family-centered and identifying social supports. Future research in larger and more diverse samples should investigate modifiable child, caregiver, and family factors as potential targets for family-focused nursing interventions.

## Supplemental Material

sj-docx-1-jfn-10.1177_10748407251333201 – Supplemental material for The Multidimensional Factors That Influence the Family Management of Autism Spectrum Disorder: A Mixed Methods Study Supplemental material, sj-docx-1-jfn-10.1177_10748407251333201 for The Multidimensional Factors That Influence the Family Management of Autism Spectrum Disorder: A Mixed Methods Study by Shayleigh Dickson Page, Kylie Trone, Margaret C. Souders, Jennifer A. Pinto-Martin and Janet A. Deatrick in Journal of Family Nursing
