# Supplementary material for: The Multidimensional Factors That Influence the Family Management of Autism Spectrum Disorder: A Mixed Methods Study
Source: J Fam Nurs. 2025 May 12;31(3):208–27. doi: 10.1177/10748407251333201 (PMC12379033; doi:10.1177/10748407251333201)
Supplement: sj-docx-1-jfn-10.1177_10748407251333201 – Supplemental material for The Multidimensional Factors That Influence the Family Management of Autism Spectrum Disorder: A Mixed Methods Study [file sj-docx-1-jfn-10.1177_10748407251333201.docx]

# INTERVIEW GUIDE

**INTRO/CONSENT TO RECORD**:

Thank you so much for participating in our study! We received your signed consent form at the start of this study, but I want to confirm your consent for this interview. We are doing this study to learn more about how families manage autism behaviors in the home setting. Participating in this interview if voluntary. You can skip any questions you do not want to answer. You can stop the interview at any time and for any reason. This interview will be recorded, but any names or places you refer to will be changed when we transcribe it so that no one will be able to identify you or your family.

Do you have any questions about the study before we get started?

Do you agree to participate in this interview?

Do I have your permission to begin recording?

| Question | Potential Probes |
| --- | --- |
| Introductory Questions | |
| Tell me about (name)  What is a typical day like for (name)?   - How is this different in the summer/school year?   Tell me about your family | - What are your child’s strengths? - What is challenging for them? - How would you describe their day compared to other kids their age? - What does your family like to do together for fun? |
| Family Management of ASD | |
| What does autism/spectrum/neurodiversity mean to you?  What does it mean to your family?  How does your child’s autism/spectrum/neurodiversity affect your role as their (mother, father, etc)? How does it affect your family?  Tell me about a recent challenge you had as their (mother, father, etc)  What does your child need the most support with? How do you help your child with that?  Tell me about a time that you felt uncertain about how to help your child with that.  How do you think your approach to caring for (name) compares to their caregivers? | When things get really challenging or overwhelming, what do you do?  What do you and other caregivers do similarly? What do you do differently?  How do you feel about your ability to help your child with (support need)?  What kinds of things influence how your feel about your ability to help your child with (support need)? |
| Feeding | |
| What happens at mealtime for your family?  (Or tell me about dinner last night)  What kinds of things do you have to think through at mealtime?  Tell me about a recent mealtime that was challenging.  What parts of mealtime do you feel that you manage well?  On the feeding questionnaire, you noted (issue), tell me more about that. | How does (mealtime issue) affect your family?  How do you decide what to do when mealtime is challenging?  How did you learn to manage it that way?  What does your family do to help your child with that?  How do you feel about your ability to help with that? |
| Sleeping | |
| What happens at bedtime at your home? (Or tell me about bedtime last night)  What kinds of things do you have to think through when putting your child to bed?  Tell me about a recent bedtime/night that was challenging.  Tell me about the parts of bedtime that you feel you manage well.  Tell me about your child’s sleep (or a specific issue on the CSHQ)  What things do you think affect the way that your child sleeps? | How does ( sleep issue) affect your family?  How do you decide what to do when your child is having difficulty sleeping?  How did you learn to manage it that way?  What does your family do to help your child with that?  How do you feel about your ability to help with that? |
| Future | |
| What are your goals for your child in the future?  What are your goals for your family in the future? |  |
